# Tender and swollen joint counts are poorly associated with disability in chikungunya arthritis compared to rheumatoid arthritis

**DOI:** 10.1038/s41598-021-98164-9

**Published:** 2021-09-17

**Authors:** Hugh Watson, Ramão Luciano Nogueira-Hayd, Maony Rodrigues-Moreno, Felipe Naveca, Giulia Calusi, Karol Suchowiecki, Gary S. Firestein, Gary Simon, Aileen Y. Chang

**Affiliations:** 1 Department of Virology, Evotec ID, Bioaster Building, 40 Avenue Tony Garnier, 69007 Lyon, France; 2 Departments of Clinical Pharmacology, Hepatology and Gastroenterology, Aarhus University, Aarhus, Denmark; 3 Laboratorio de Parasitologia e Vetores da Amazonia, Curso de Enfermagem, Centro de Ciencias da Saude, Universidade Federal de Roraima, Boa Vista, RR Brazil; 4 Laboratório de Ecologia de Doenças Transmissíveis na Amazônia, Instituto Leônidas e Maria Deane, FIOCRUZ, Manaus, Brazil; 5 Biometry Department, Aptuit (Verona) Srl, Verona, Italy; 6 Department of Medicine, George Washington University, Washington, DC USA; 7 Department of Medicine, University of California San Diego, San Diego, CA USA; 8 School of Medicine and Health Sciences, George Washington University, Washington, DC USA

**Keywords:** Viral infection, Rheumatoid arthritis, Outcomes research

## Abstract

Chronic rheumatological manifestations similar to those of rheumatoid arthritis (RA) are described after chikungunya virus infection. We aimed to compare the relevance of joint counts and symptoms to clinical outcomes in RA and chronic chikungunya disease. Forty patients with chronic chikungunya arthralgia and 40 patients with RA were enrolled in a cross-sectional study. The association of tenderness and swelling, clinically assessed in 28 joints, and patient evaluations of pain and musculoskeletal stiffness with modified Health Assessment Questionnaire (HAQ) and quality of life (QoL) assessments were investigated. Tender and swollen joint counts, pain and stiffness scores were all associated with the HAQ disability index in RA (all r > 0.55, *p* ≤ 0.0002), but only stiffness was significantly associated with disability in chikungunya (r = 0.38, *p* = 0.02). Joint counts, pain and stiffness were also associated with most QoL domains in RA patients. In contrast, in chikungunya disease, tender joint counts were associated only with one QoL domain and swollen joints for none, while pain and stiffness were associated with several domains. Our results confirm the relevance of joint counts in RA, but suggest that in chronic chikungunya disease, joint counts have more limited value. Stiffness and pain score may be more important to quantify chikungunya arthritis impact.

## Introduction

Infection with chikungunya virus (CHIKV) is typically announced by a fever accompanied by a rapid onset of painful and swollen joints^1^. The fever and joint involvement often resolve after 7–10 days, but in an estimated 40% (95% CI 31–49) of patients painful and stiff joints persist for months or years^2,3^. The chronic joint pain and inflammation occurring after infection with chikungunya virus is less well characterized than rheumatoid arthritis (RA), but shares many of the same features, such as pain and swelling in multiple joints accompanied by stiffness with the smaller joints of the upper and lower limbs most commonly affected^4–6^. Instruments used for evaluating clinical status in RA may therefore also be useful in CHIKV arthritis.

The rheumatologist has many different instruments at his disposal to evaluate the severity of disease in a patient with RA, instruments which have been extensively tested and proven to be of value. Tender and swollen joint counts are well established in RA as standard measures of joint involvement^7^, and recent work has addressed the comprehensive assessment of joint stiffness in RA, a previously understudied area^8,9^. These same measures and instruments have also been employed in CHIKV arthritis studies, but their usefulness in chikungunya disease is less clear^10^.

As chikungunya outbreaks have become more widespread, threatening not only tropical, but also semi-tropical and some temperate regions of the world, there is an increasing need to identify effective therapeutics for chronic chikungunya disease. It is important, therefore, to know whether the instruments used in RA are equally appropriate for the assessment of post-chikungunya arthritis.

Following an outbreak of CHIKV infections from the CHIKV ECSA (East, Central and South African) genotype in Roraima State, Brazil, our group has reported a cross-sectional study comparing the characteristics of patients with post-chikungunya arthritis with three different local control groups: patients without chronic arthritis following chikungunya infection, chikungunya negative patients with RA and healthy subjects^11^. We reported arthritis severity 2 years after CHIKV infection similar to that in the patients with RA. Chronic chikungunya disease, like RA, results in measurable disability and significantly impaired quality of life^12,13^. Counts of involved joints have been shown to correlate well with these patient-reported outcome measures in RA^14–17^, but based on our own previous work in chikungunya disease^10^, we hypothesized that the relationship of these disease measures to patient-reported outcomes may differ in the two diseases. In the present analysis, we further explored the relationship of disease and symptom severity measures with patient-reported outcomes in RA and CHIKV arthritis, with the objective of determining whether joint counts and symptom measures are associated with disability and health-related quality of life (HRQoL) in a similar manner in RA and in patients with post-chikungunya arthritis.

## Patients and methods

### Study design

This was a cross-sectional analysis investigating the relationship of arthritis severity measures to patient outcomes in two groups of patients with arthritis of different origins.

### Setting and participants

The study was conducted in Boa Vista, capital city of Roraima State in northern Brazil. This area was the scene of a large outbreak of chikungunya virus infections associated with the ECSA genotype of the virus, whose emergence in Roraima is estimated to date from mid-2016^18^. Between June and August 2019, two to three years after the outbreak, 40 patients with chronic chikungunya arthritis and 40 CHIKV-negative patients with RA were enrolled into the study. Chikungunya virus (CHIKV) cases were identified by the sudden onset of fever and severe arthralgia or acute onset arthritis in a person residing in or travelling to an endemic or epidemic area within 14 days prior to symptom onset. CHIKV infection was confirmed at the Roraima Central Laboratory/Laboratório Central do Estado de Roraima (LACEN) using RT-qPCR. The diagnoses of arthritis in chikungunya patients and rheumatoid arthritis were made by rheumatologists at Coronel Mota Hospital. Rheumatoid arthritis was diagnosed using the American College of Rheumatology (ACR) criteria^19^.

The study protocol was approved by the Committee for Ethics and Research at the Federal University of Roraima (Protocol number 3.406.0703). All research procedures were followed in accordance with the Helsinki Declaration of 1975, as revised in 1983. All patients gave informed consent prior to their inclusion in the study.

### Clinical evaluation and outcome measures

Patients were assessed by a rheumatologist at a face-to-face interview. Twenty-eight joints were assessed for tenderness and swelling. The specified 28-joint counts of tender and swollen joints used have been validated with respect to more comprehensive joint counts in RA and are now a widely used standard in this indication^20–22^. Symptoms were evaluated by the patient with a pain intensity visual analogue scale (0–100) and a 21-item musculoskeletal stiffness questionnaire (overall score expressed as a percentage) which incorporated several aspects of stiffness severity as well as the physical and psychological impacts of stiffness^9,23^. Functional disability was assessed with the modified Stanford Health Assessment Questionnaire (HAQ). This questionnaire is widely used in rheumatologic diseases and asks the degree of difficulty experienced by subjects in a number of daily activities, with responses consolidated to generate an overall score between zero for no disability and three for maximum disability^24^. The Euroqol EQ5D-5L questionnaire, a generic health-related quality of life instrument used in many areas of health outcomes research, was employed to measure the patients’ quality of life^25^. The EQ5D-5L assesses quality of life in five domains: mobility, self-care, usual activities, pain-discomfort and anxiety-depression.

### Statistical analysis

Symptoms, scores and joint counts in the two groups were compared using the Mann–Whitney non-parametric test. Associations of joint pain, joint stiffness, tender joint count and swollen joint count with the HAQ disability score and the five domains of the EQ5D-5L quality of life instrument were analysed using Spearman’s rank correlation test for each disease individually. Regression analysis was performed to determine whether there was a statistically significant difference between the associations in RA and in CHIKV arthritis.

### Ethics approval

The study protocol was approved by the Committee for Ethics and Research at the Federal University of Roraima (Protocol number 3.406.0703). All research procedures were followed in accordance with the Helsinki Declaration of 1975, as revised in 1983. All persons gave informed consent prior to their inclusion in the study. No animals were used in this study.

### Consent to participate

All research procedures were followed in accordance with the Helsinki Declaration of 1975, as revised in 1983. All persons gave informed consent prior to their inclusion in the study.

### Consent for publication

Not applicable.

## Results

### Demographic and clinical characteristics of participants

In both the RA and CHIKV arthritis groups the patients were predominantly female (93% and 90%, respectively), with an education level at least of secondary school (in 65% and 58%, respectively) and median ages of 51.5 years (interquartile range 40.5–62) and 49.5 years (interquartile range 42–55), respectively. Patients were of 85% and 88% Pardo (mixed) ethnicity, the majority ethnic group in Roraima State, in the RA group and CHIKV arthritis group, respectively. The chikungunya patients were assessed a median of 27 months (interquartile range 22.5–29) after infection. None of this group had reported arthritis prior to chikungunya infection.

The RA and CHIKV arthritis patients had similar symptom scores for pain intensity and stiffness severity, and similar levels of disability as measured by the HAQ (Table 1). However, compared to the RA patients, the CHIKV arthritis patients had significantly fewer swollen joints and a slightly smaller number of tender joints on average (Table 1). The use of medications for arthritis at the time of clinical evaluation also differed significantly with more patients in the RA group taking steroids (60%) or methotrexate (40%) compared to the CHIKV arthritis group (15% and 5% respectively).

**Table 1:** Disease characteristics of patients with rheumatoid and chikungunya arthritis.

Severity or symptom measure	Rheumatoid arthritis (n = 40)	CHIKV arthritis (n = 40)	*p* value
Tender joint count	14 (7–24)	12 (4–14)	0.03
Swollen joint count	12 (3–24)	3 (0–12)	0.002
Joint pain (VAS)	75 (50–90)	73 (50–82)	0.97
Stiffness severity (%)	55 (32–64)	54 (30–61)	0.95
HAQ	0.56 (0.25–1.25)	0.50 (0.25–0.75)	0.22

Results expressed as median (interquartile range).

### Association of joint counts and symptoms with clinical outcomes

**Figure 1:**
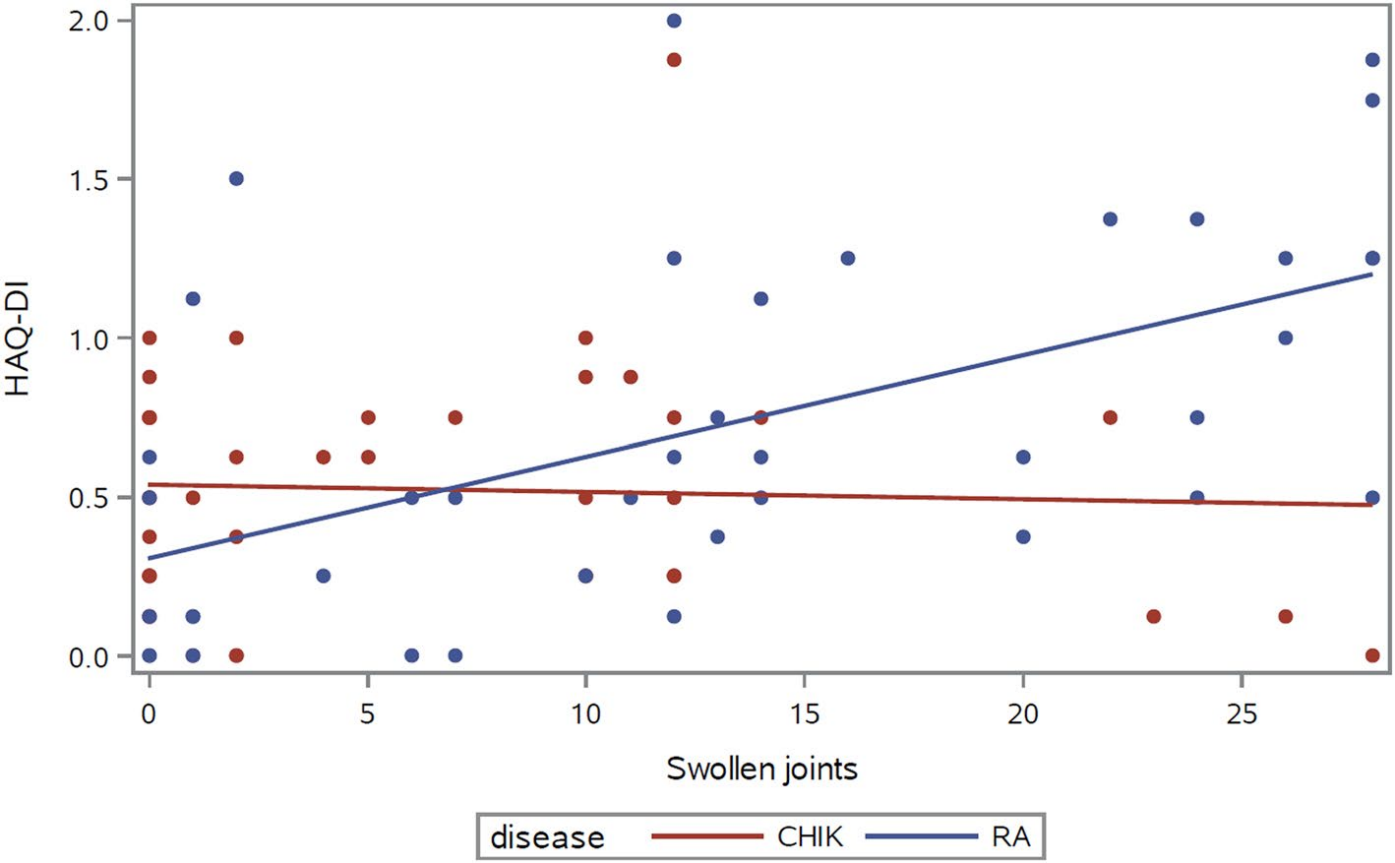
Relationship of swollen joint count to the HAQ-disability index in rheumatoid arthritis and in chikungunya arthritis; *p* = 0.003 for the interaction disease*swollen joint count.

Tender and swollen joint counts, pain and stiffness were all correlated with the HAQ disability index in RA, but only stiffness was significantly associated with disability in chikungunya patients (Table 2). Regression analysis revealed a statistically significant interaction term between the disease diagnosis and swollen joint count, confirming that the effect of swollen joint count on disability significantly changes between RA and CHIKV arthritis (*p* = 0.003 for the interaction of diagnosis with swollen joint count) (Fig. 1). For tender joint counts, pain intensity and stiffness, the interaction term with disease showed a similar trend without reaching statistical significance (0.05 < *p* < 0.10).

**Table 2:** Association of disease severity with HAQ disability index in rheumatoid and chikungunya arthritis.

Severity or symptom measure	Rheumatoid arthritis (n = 40)	Chikungunya arthritis (n = 40)
	r (*p*)	r (*p*)
Tender joint count	0.56 (0.0002)	0.24 (0.14)
Swollen joint count	0.60 (< 0.0001)	0.002 (0.99)
Joint pain (VAS)	0.55 (0.0002)	0.29 (0.07)
Stiffness severity	0.57 (0.0001)	0.38 (0.02)

Results expressed as Spearman’s rank correlation coefficients.

**Figure 2:**
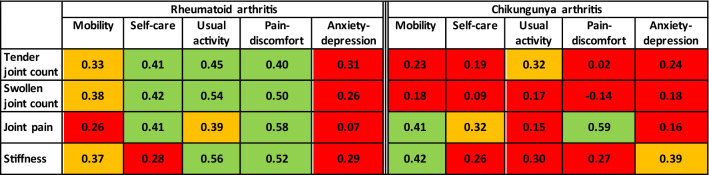
Association of disease severity with EQ5D quality of life domains in rheumatoid and chikungunya arthritis. Spearman’s Rank Correlation coefficient: red indicates not significant, orange indicates *p* < 0.05 and green indicates *p* < 0.01.

**Figure 3:**
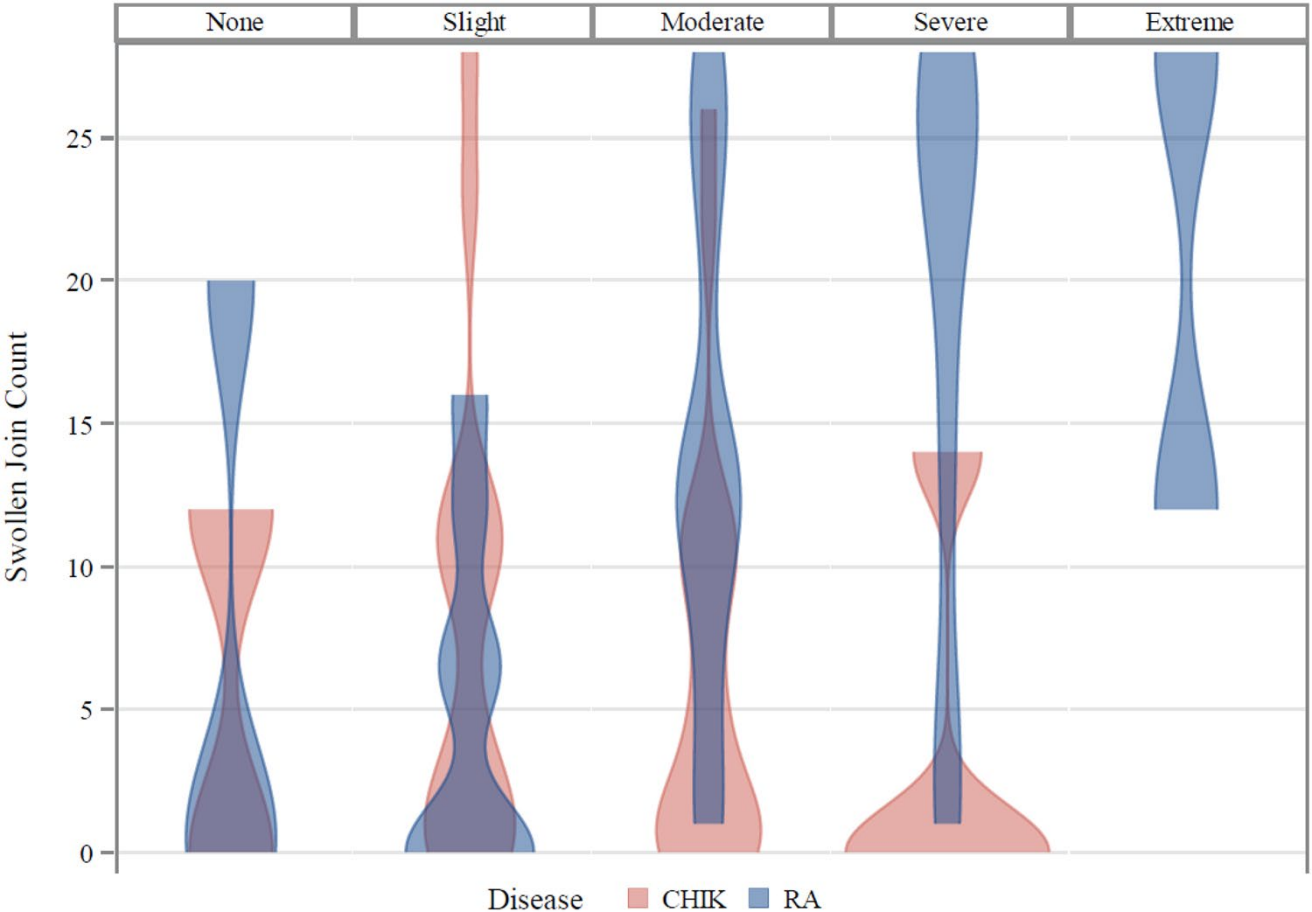
Swollen joint counts shown by EQ5D level of pain/discomfort for chikungunya arthritis (CHIK) and rheumatoid arthritis (RA); *p* = 0.006 for the interaction disease*swollen joint count in multiple regression analysis.

Tender and swollen joint counts, pain and stiffness were all associated with the majority of EQ5D quality of life domains (except anxiety/depression) in RA patients (Fig. 2). In contrast, in CHIKV arthritis, tender joint counts were significantly correlated (*p* < 0.05) only with one quality of life domain, while pain intensity score correlated with impaired mobility, self-care and pain/discomfort domains, and stiffness was associated with the mobility, usual activity and anxiety/depression domains (Fig. 2). Swollen joint counts were not significantly correlated with any of the quality of life domains in chikungunya disease. Regression analysis further confirmed that the effect of swollen joint count on the pain/discomfort domain was significantly different in RA and CHIKV arthritis (*p* = 0.006 for the statistical interaction of underlying disease with number of swollen joints), the degree of pain and discomfort increasing with the number of swollen joints only in RA patients (Fig. 3).

## Discussion

Chronic post-chikungunya rheumatism, like rheumatoid arthritis, has a large impact on HRQoL^13,26–28^ and results in a moderate disability even more than two years after infection^12^. The present study compared patients with CHIKV arthritis 2–3 years post-infection with a group of RA patients who had similar levels of self-reported joint pain and stiffness and similar levels of disability. In the patients with RA, we were able to confirm the well-established strong association of tender and swollen joint counts and patient-reported symptoms with disability using the HAQ disability index^14–16^ and with HRQoL using the EQ-5D^17^. In contrast, in the patients with CHIKV arthritis, only patient-reported symptoms were clearly associated with the disability and HRQoL instruments. Despite the patients with CHIKV arthritis having only slightly fewer tender joints, the association of tender joint counts with disability and HRQoL was markedly weaker than that in RA.

The weak relationship of swollen joint counts with both disability and quality of life in post-chikungunya rheumatism was observed in a previous study, in which patients had very low numbers of swollen joints^13^. In the present study, swollen joints were more frequent, but the weak relationship of swollen joint count to patient-reported outcomes in chronic chikungunya disease was confirmed. Pain and stiffness occurring in the absence of notable swelling may be indicative of subclinical synovitis, a finding which has been reported following ultrasound examination of joints in patients with chronic musculoskeletal symptoms after chikungunya infection^29^. Incorporation of musculoskeletal ultrasound in the assessment of chronic chikungunya may help to explain the impact of the disease on function and quality of life.

Self-report scales in rheumatology commonly intercorrelate well^30^, so the association of patients’ subjective assessment of symptoms (e.g. pain and stiffness) with HRQoL scores might be expected to be stronger than that seen with more objective disease measures (e.g. joint scores or serum biomarkers of inflammation). Patients’ perceptions may influence in a similar fashion reporting of symptoms and outcomes, and this may be accentuated in the case of affliction by a poorly understood disease such as chikungunya. The potential role of subjectivity in reporting symptoms, influenced by the preceding intense acute fever has been discussed in this disease^31^. In this scenario, many symptoms and disabilities may be attributed to chikungunya. Indeed, compared to the joint counts, the self-assessment of symptom severity by the patients with CHIKV arthritis in the present study was more closely related to disability and some quality of life domains. This was particularly true for joint stiffness, which seems to be a frequent and relevant symptom in chikungunya patients deserving careful assessment. However, even allowing for this explanation of joint counts correlating less strongly than symptom scores with patient-reported outcomes, the association of joint counts with outcomes in patients with CHIKV arthritis was notably weak compared to our findings in the RA group.

There are certain limitations of the present study, of which one was the restriction of the clinical examination to 28 joints. Although a commonly used standard in recent RA studies, this 28-joint count excludes the more distal joints of the lower limb, joints which are frequently involved in chikungunya disease^32^. This opens the possibility that involvement of certain joints not assessed in this study may have had an important impact on disability and some quality of life domains. It would be of interest to re-investigate the association of joint counts with patient outcomes using counts expanded to include the more distal lower limb joints. It may also be important to consider that joint counts evaluated here offer only a snapshot at a single time-point and that post-CHIKV musculoskeletal disorders can result in a variety of phenotypes ranging from rapidly resolving inflammatory disease to chronic inflammatory rheumatic disorders and include those with a relapsing–remitting pattern of symptoms, which may be inconsistently captured by a physical examination.

Matching groups of patients with RA and chikungunya arthritis for such a study is not straightforward. Our groups were well-matched demographically, which is important in comparing joint stiffness^33^, and well matched for symptom severity and for disability. However, there were large differences in swollen joint counts and use of medications. We also had incomplete information on the disease history of RA and no possibility to compare objective serum markers. The course of the two diseases is quite different: RA is progressive, while joint symptoms in chikungunya patients are extremely disabling in the acute phase and generally resolve slowly over time^2,34^, so matching patients for duration of disease would not be meaningful. Erythrocyte sedimentation rate (ESR) and C-reactive protein (CRP) are well-characterised in RA, but ESR is not elevated in chikungunya patients and CRP levels are primarily elevated during the acute phase of infection^35^, thus limiting any opportunity for matching the patient groups on these objective laboratory parameters. There is also no strong evidence-base yet for the treatment of chronic CHIKV arthritis, so certain differences between the groups in disease history and use of disease-modifying drugs for arthritis were to be expected. Nonetheless, the similarities in the symptom and disability scores suggest that our groups were adequately matched in terms of disease impact for the purpose of this study.

In terms of the generalizability of our findings, two further aspects should be considered. Inter-genotype heterogeneity in the prevalence of chronic post-CHIKV symptoms has been suggested^36^. Our post-CHIKV cohort had most probably been exposed to an ECSA lineage circulating in this part of Brazil^18^. Although our earlier findings in a Colombian cohort exposed to the Asian genotype were broadly in line with the present study^10^, it cannot be excluded that the pattern and importance of joint inflammation may differ from patients infected with the Indian Ocean lineage. Secondly, considering the subjective nature of self-reported outcome measures, evaluation bias can play a role and may differ between populations depending on the cultural context. As both the present study and our previous work was performed in South America, confirmation in Asian or African populations would reinforce the conclusions which can be drawn.

Defining the most relevant measures of disease severity in chronic chikungunya rheumatism needs further study. No single measure of disease severity tested here seems to predict very well the impact of chikungunya disease on the patient. Although musculoskeletal manifestations feature prominently in chronic chikungunya disease, other systemic manifestations such as asthenia, fatigue, anxiety and depression are reported^4,31,37,38^. These are also likely to be associated with HRQoL in chronic chikungunya rheumatism, possibly explaining why measures of joint involvement alone were so poorly predictive of HRQoL in our study. While instruments designed for use in rheumatoid arthritis provide a good starting point in the assessment of CHIKV arthritis, the construction of a disease severity index incorporating additional variables adapted to post-chikungunya rheumatism may be of value in the future study of this condition and in identifying the most effective treatments. Future research efforts in this direction should therefore take into account the impact of chronic fatigue, depression and other neurosensory sequelae of chikungunya infection in order to capture the full burden of the disease.

## Data Availability

The datasets generated during and/or analysed during the current study are stored in the REDCap database at The George Washington University. Datasets may be available on reasonable request.
